# *Funalichnus bhubani* isp. nov. from Bhuban Formation, Surma Group (Lower -Middle Miocene) of Aizawl, Mizoram, India

**DOI:** 10.1371/journal.pone.0077839

**Published:** 2013-10-18

**Authors:** Raghavendra Prasad Tiwari, Chinmoy Rajkonwar, Satish Jaychandbhai Patel

**Affiliations:** 1 Department of Geology, Mizoram University, Aizawl, Mizoram, India; 2 Department of Geology, Faculty of Science, the Maharaja Sayajirao University, Baroda, Vadodara, Gujarat, India; The Evergreen State College, United States of America

## Abstract

A new ichnospecies of the ichnogenus *Funalichnus* Pokorný is described from the Middle Bhuban Unit, Bhuban Formation, Surma Group (Lower - Middle Miocene) of Aizawl, Mizoram, India. *Funalichnus bhubani* isp. Nov. Is a large burrow displaying cylindrical segments that are oriented nearly perpendicular to the bedding plane. The new ichnospecies can be identified on the basis of general form, size, unlined passive filling and twisted rod-like structure. The association of *Funalichnus bhubani* isp. Nov. With *Arenicolites*, *Diplocraterion*, *Ophiomorpha Psilonichnus Skolithos* and *Thalassinoides* points to its bathymetric restriction. The deep extension of the burrow in clastic sediments provides a favourable condition for preservation in the shoreface environment and occurrence in fine- to medium-grained clastic sediments may be a preservational preference.

## Introduction

Fritsch [1] described ichnospecies *Fucoides strangulates* from the Upper Cretaceous of the Bohemian Basin, Czech Republic. Later, Pokorný [2] studied museum collections of Fritsch [1] and additional materials from the type localities of the Bohemian Basin and re-designated it as a new ichnogenus *Funalichnus* and its type ichnospecies *Funalichnus strangulates* [2].

The purpose of the paper is to describe a new ichnospecies *Funalichnus bhubani* from the Middle Bhuban Unit of the Bhuban Formation, Surma Group (Lower - Middle Miocene) of Aizawl, Mizoram, India. Environmental and preservational aspects of the new ichnospecies are discussed to refine the ichnofacies association. Earlier records of trace fossils from the Cenozoic succession of Mizoram include *Palaeophycus* from Barail succession [3], *Teredolites clavatus* from Upper Bhuban Unit of Bhuban Formation [4], and a diversified assemblage belonging to *Skolithos* and *Cruzian*a ichnofacies from the Middle and Upper Bhuban Units of the Bhuban Formation [5,6].

## Location and Geology

The sedimentary succession of Mizoram has been geologically subdivided into the Barail (Oligocene), the Surma (Lower to Middle Miocene) and the Tipam groups (Upper Miocene to early Pliocene) in ascending order [7,8]. The nearly 8000 m thick rock succession exposed in the Mizoram state, India, comprises a series of N-S trending elongated folds having a sub-meridional trend and arcuate shape with westward convexity. The Mizoram area has ~6000 m of exposed Surma succession constituting the depocentre for The Surma Group. This group consists of a repetitive succession of arenaceous and argillaceous rocks with a few intercalations of shell limestone, calcareous sandstone and intraformational conglomerate [8]. It has been subdivided into the Bhuban and the Bokabil Formations. The Bhuban Formation is the thickest lithostratigraphic unit in the Mizoram State attaining ~5000 m of thickness and is further subdivided into Lower, Middle and Upper Bhuban Units. The main lithologies of Bhuban Formation are sandstone, siltstone, shale, mudstone and their admixtures in various proportions. The ages of these stratigraphic units have been constrained on the basis of the fossil contents [9–13].

**Figure 1:**
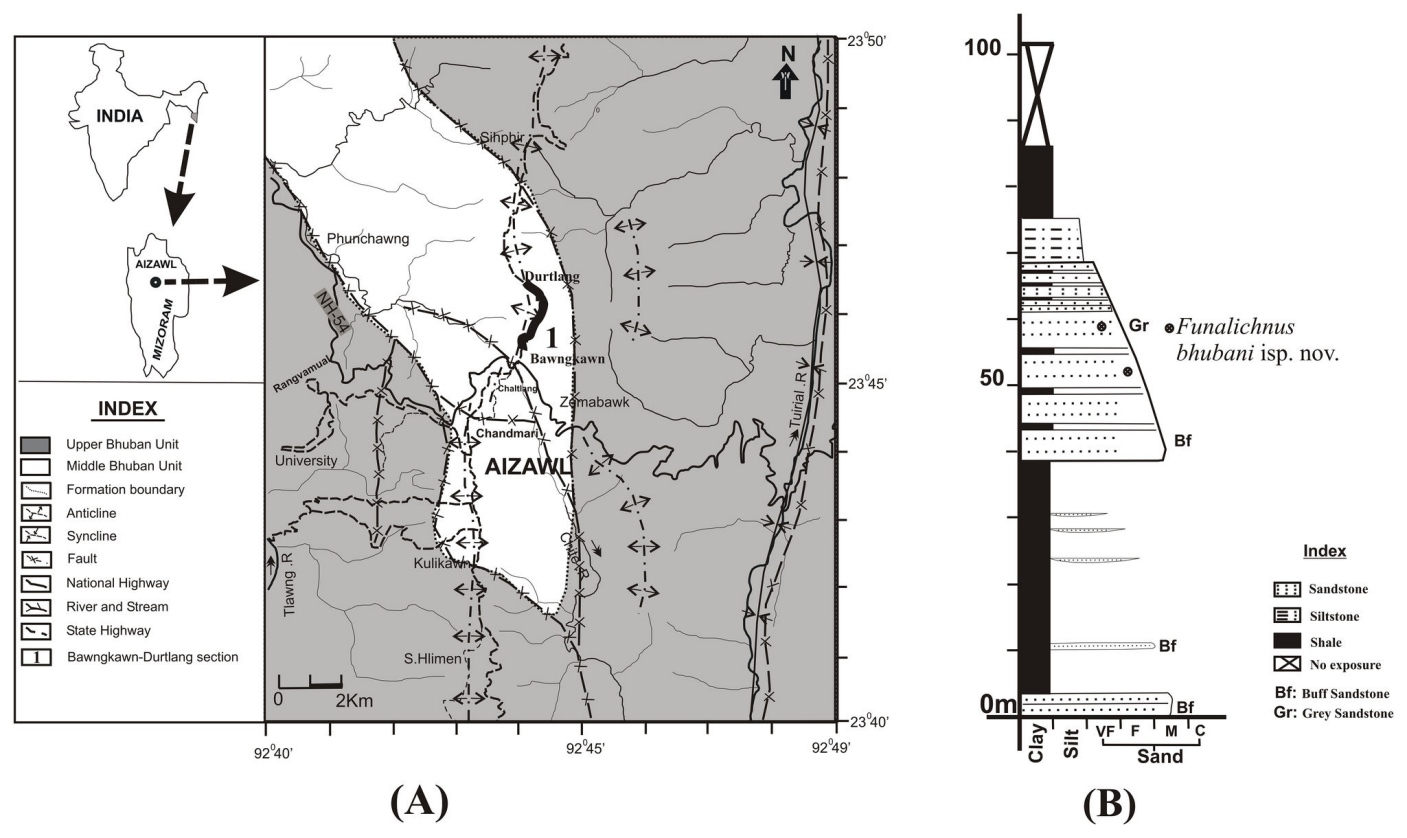
Study Area (a) Location and geological map. (B). Litho-column of a part of Bawngkawn-Durtlang section showing trace fossil yielding silty-sandstone bed.

The material described in this paper comes from the Bawngkawn-Durtlang road section (latitude 23°45ʹ21.9ʺ N to 23°46ʹ10.5ʺ N and longitude 92°43ʹ51.9ʺ E to 92°44’08ʺ E) on the northern part of Aizawl city, Mizoram, North East India (Figure 1). Nearly 615 m of rock succession belonging to the lower part of the Middle Bhuban Unit of the Bhuban Formation, Surma Group (Lower - Middle Miocene) age is well exposed in this section. The Middle Bhuban Unit in this section comprises a succession of sandstone, silty-sandstone, siltstone, silty-shale, shale, sandy-shale and mudstone. Fine- to medium-grained grey- and brown-coloured sandstone of this unit is bioturbated and bears a diverse assemblage of trace fossils [6]. The new ichnospeices described in this report comes from grey-coloured sandstone exposed at latitude 23°45’54.2” N and longitude 92°44’04” E. Specific permission was not required to collect fossils from this locality as it is not a part of a national park or other protected area of land.

**Figure 2:**
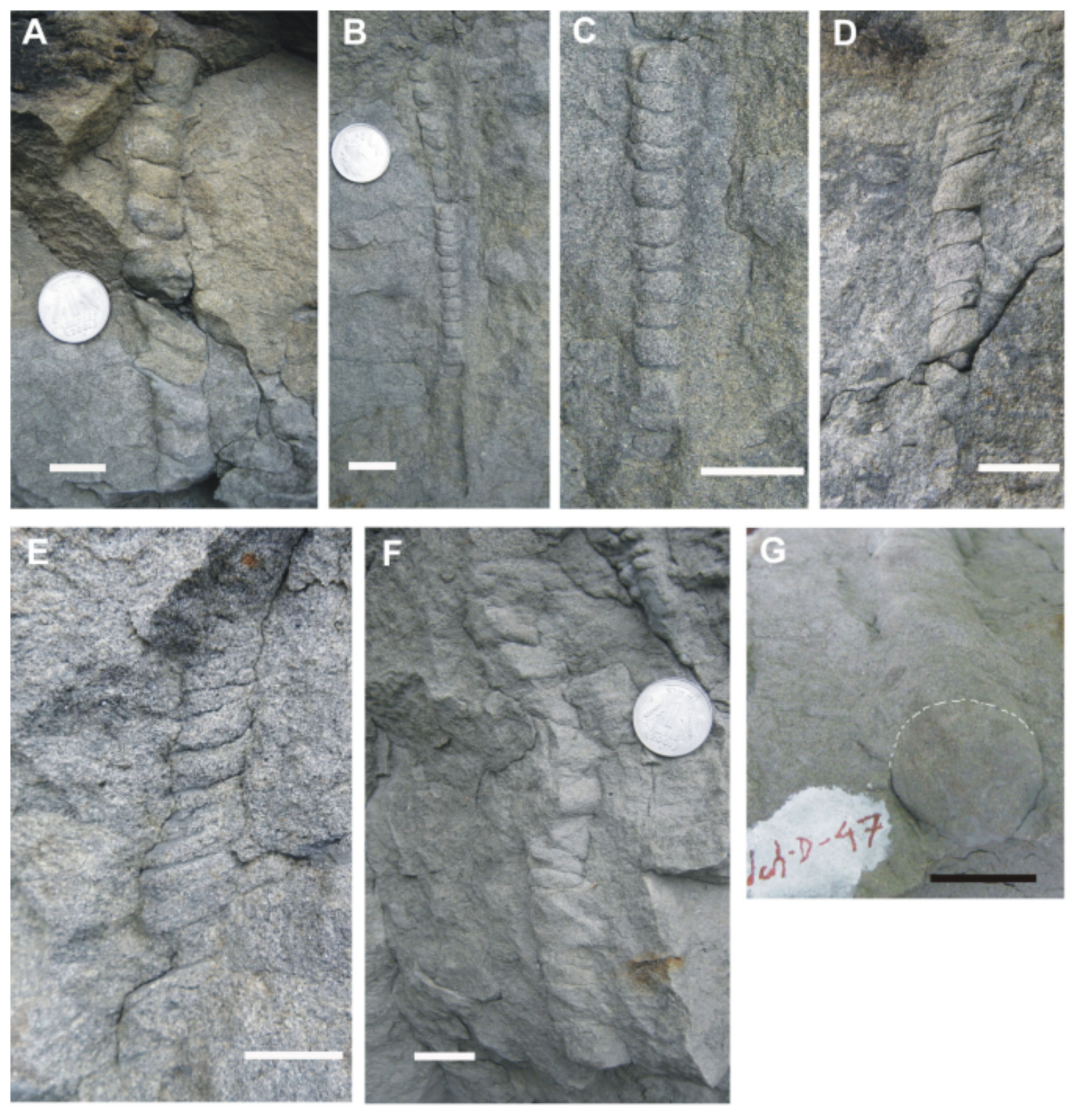
Photographs of *Funalichnus bhubani* isp. **nov**. Scale bar in every figure is 20 mm. A. *Funalichnus bhubani* isp. nov., holotype, vertical burrow consisting of cylindrical segments with uniform dimensions, lower part shows variation in segment direction. (Registration No. Ich/D/46). B. *Funalichnus bhubani* isp. nov., *Paratype*, vertical burrow with uniform segments. Upper and lower parts are poorly preserved. C. *Funalichnus bhubani* isp. nov., *Paratype*, close-up of Figure 2B shows equi-dimensional segments with horizontal shallow interspaces. *Note*: Upper and lower parts of the burrow are broken. D. *Funalichnus bhubani* isp. nov., *Paratype*, steeply inclined burrow shows variable dimension of the cylindical segments and interspaces are inclined towards the left side. E. *Funalichnus bhubani* isp. nov., *Paratype*, steeply inclined burrow shows variable dimension of the segments and interspaces are inclined towards the left side. F. *Funalichnus bhubani* isp. nov., *Paratype*, steeply inclined and gently bend showing more irregular forms of segments. Interspaces are inclined towards the right side (Registration No. Ich/D/47). G. Cross section of Figure 2F showing circular outline.

## Systematic Ichnology

**Ichnogenus:** *Funalichnus* Pokorný, 2008.

**Type ichnospecies:** *Funalichnus strangulatus* Fritsch, 1883.

**Amended Diagnosis** Sub-vertical to vertical, straight, simple burrow, ovoid in cross-section, smooth, ornamented with closely spaced, cylindrical segments oriented obliquely to the axis. Lower termination of the burrow is smooth and tapering. Burrow fill is structureless, homogenous and finer than the surrounding rock.

**Ichnospecies** *Funalichnus bhubani* isp. nov.

Figure 2 (A-F).

**Material:** Four sandstone slabs (Ich/D/46, 47, 48 and 49) and four field photographs housed in the Palaeontology Laboratory of the Department of Geology, Mizoram University, Aizawl-796 004, Mizoram, India.

**Holotype:** A sandstone slab bearing registration no. Ich/D/46 (Figure 2A).

**Paratypes:** Sandstone slabs bearing specimen nos. Ich/D/47, 48 and 49 and four field photographs (Figures 2B-F). 

**Type Locality:** Bawngkawn-Durtlang road section, Aizawl, Mizoram, North East India (latitude 23°45ʹ54.2ʺ N and longitude 92°44ʹ04ʺ E).

**Type Horizon:** Fine- to medium-grained, grey-coloured and bioturbated sandstone bed of Middle Bhuban Unit, Bhuban Formation, Surma Group (Lower to Middle Miocene). 

**Etymology:** The specific name is derived from the occurrence of this ichnospecies in the Bhuban Formation of Mizoram, India.

**Diagnosis:** Long, smooth, cylindrical, unlined, unbranched, vertical or steeply inclined burrow ornamented with closely spaced, distinct, cylindrical segments oriented either perpendicular or slightly oblique to the axis. Dimensions of segments are mostly uniform but vary slightly along the length. 

**Description:** Endichnial, long, unbranched, vertical to steeply inclined, straight to gently curved and unlined burrow. The burrow consists of a number of small cylindrical segments imparting a twisted rod like structure to the burrow. The interspaces between the cylindrical bodies form curved depressions. Cylindrical bodies of the burrow mostly have uniform dimensions (Figure 2A and 2B) but also show slightly variable dimensions along the length (Figure 2C) and occasionally also display irregular form (Figure 2E). The individual bodies are smooth and are slightly higher as compared to the interspaces, which are usually parallel to the bedding plane (Figure 2A and B) and are inclined to right or left sides (Figures 2A, 2C, 2D and 2E). Cross-section of shaft is circular to sub-circular in outline. Maximum observed length of the burrow is 200 mm (Figure 2B) whereas diameter of the burrow varies from 10 to 36 mm (Figure 2D and 2E). Burrow fill is identical to the host sediments.

**Remarks:** *Funalichnus*, as a new ichnogenus was described by Pokorný [1] from the Upper Cretaceous of the Bohemian Basin, Czech Republic, and includes the type ichnospecies *Funalichnus strangulatus*. Earlier, it was described as *Hamites intermedius* [14,15], *H. strangulatus* [16,17] and *Fucoides strangulatus* [2]. Pokorný [1] described the ichnogenus *Funalichnus*, based on the type material and the additional materials from the subsequent collections from the Upper Cretaceous of the Bohemian Basin, as the small burrow with a maximum length of 51 mm that is characterized by closely spaced swollen ribs with lower termination of the burrow tapering downwards to one side and smooth on the surface.

*Funalichnus bhubani* isp. nov. differs from the type ichnospecies of Pokorný [1] in general morphologic features and dimensions; it is relatively a large burrow with observed length in the range of 120-200 mm and diameter in the range of 10-36 mm. Cylindrical bodies are either symmetrical or asymmetrical and individual segments show slight variation in their morphology and interspaces between them are either horizontal or inclined towards the right (Figure 2A & 2F) or the left (Figure 2D & 2E) side and in some cases diameter gradually increases downwards (Figure 2E). 

*Ichnogyrus nididens* described by Bown and Kraus [18] shows tight coiling and is symmetrical forming a closed spiral in which successive whorls are in contact. It is a very large burrow; even incomplete burrow with only six preserved whorls measures 22.2 cm in length and 4.6 cm in diameter. The overall architecture of *Ichnogyrus nididens* [18] is also different from that of *Funalichnus bhubani* isp. nov.

The vertical nature and cylindrical segmented form of *Funalichnus bhubani* isp. nov. indicates that the animal excavated the surrounding compact sediments to its body length and pushed the sediments periodically downward to maintain its position. The nearly uniform nature of the segments points to the systematic work of the animal and also reveals the body size of the burrower. Periodically filled structures are interpreted as a dwelling structure that may have had some combined feeding habits. Dwelling/feeding structures like *Ophiomorpha* and *Thalassinoides* (6) are abundantly found associated with the described new ichnospecies indicating behaviourally restricted structures that may be interpreted as crustacean burrows.

## Environmental Significance

Burrows considered in this study are found in fine- to medium-grained, grey-coloured and bioturbated sandstone of Middle Bhuban Unit, Bhuban Formation, Surma Group (Lower to Middle Miocene). The lithologies exposed in this section exhibit sedimentary structures such as cross laminations/beddings, ripple marks, sole marks and load casts. *Funalichnus bhubani* isp. nov. occurs in association with *Arenicolites*, *Diplocraterion*, *Psilonichnus* and *Skolithos* which are endichnial burrows of chiefly suspension feeding organisms and are typical members of the *Skolithos* ichnofacies [19,20] The presence of the *Skolithos* ichnofacies indicates the unconsolidated and shifting nature of the substrate, high energy conditions and a rapid change in the sedimentation rate and erosion of surface sediments [21,22]. The association of *Funalichnus bhubani* isp. nov. with other trace fossils [6] thereby reflects the importance of the local behavioural changes of the trace maker in environmental conditions that prevailed during deposition of the host rocks. Thickly-bedded grey-coloured silty-sandstone facies of the same age that are located near the Durtlang area in Mizoram, India bear *Psilonichnus upsilon* suggesting a backshore marginal marine environment [23]. *Ophiomorpha* and *Thalassinoides* burrows of crustaceans have been described by Rajkonwar [11] and association of *Funalichnus bhubani* isp. nov. with these burrows indicates the changes in the colonization pattern of the benthic community. Abundance of *Arenicolites, Diplocraterion, Ophiomorpha, Psilonichnus* and *Skolithos* and sedimentary characteristics may be attributed to a relatively moderate to high wave and current energy conditions and shifting substrate exploited by the opportunistic animals in the foreshore/shoreface environments. Moreover, associated ichnogenera are intimately related to high-energy shoreface environments indicating that the producer of the *Funalichnus bhubani* isp. nov. also occupied a similar type of environmental setting. The ichnofossils of the *Skolithos* ichnofacies are produced by suspension feeders and are associated with a relatively high level of wave or current energy, typically developed in a clean well-sorted loose or shifting substrate [22]. These conditions commonly occur at or near the shoreface and sheltered foreshores but similar conditions also occur in a wide range of high-energy shallow-water environments [23,24]. The deeper extension of burrows belonging to this new ichnospecies in a fine- to medium-grained clastic sediments favoured its preservation. 
